# A systematic review of educational resources for teaching patient handover skills to resident physicians and other healthcare professionals

**Published:** 2013-03-31

**Authors:** Mark F. Masterson, Richdeep S. Gill, Simon R. Turner, Pankaj Shrichand, Meredith Giuliani

**Affiliations:** 1 Canadian Association of Interns and Residents, Ottawa, Ontario, Canada; 2 Dept. of Anesthesiology and Pharmacology, University of British Columbia, Vancouver, Canada; 3 Dept. of Surgery, University of Alberta, Edmonton, Alberta, Canada; 4 Dept. of Radiation Oncology, University of Toronto, Ontario, Canada

## Abstract

**Background:**

As physicians reduce their work hours, transfer of patient care becomes more common; this is a time of heightened risk to patients. Training in patient handover skills may reduce this risk. The objective of this study was to systematically review the literature regarding education models available to teach handovers skills to healthcare professionals.

**Methods:**

Two investigators independently reviewed educational publications for inclusion/exclusion. A third reviewer resolved any disagreement. Included papers contained an educational resource for teaching handover skills to any health profession in any patient population. Papers were rated on a previously described 4 point scale for quality.

**Results:**

1746 papers were identified, of which 12 met the inclusion criteria These studies presented information on educational curricula, simulation technologies and didactic sessions. The most common educational method was simulation or role-playing, which is better received by learners than didactic sessions. Teaching handover practices makes residents feel more confident in their handover, and residents receiving adequate handover are more comfortable with their duties.

**Conclusions:**

Although data are limited, effective training models for handover skills have been described in the literature. Residents and other healthcare practitioners should receive training in handover to improve practitioner comfort and patient care.

## Introduction

According to the 2007 National Physician Survey, physicians are currently working or intend to work fewer hours.^1^ Residents’ work hours restrictions have been mandated by the various provincial contracts in Canada, in the Accreditation Council for Graduate Medical Education (ACGME) guidelines in the United states, and the European Work Time Directives, in part as a guarantee of patient safety.^2^,^3^,^4^,^5^,^6^ Reducing physician work hours increases the transfer of care of patients from one physician to another.^7^ Many authors have identified this transfer of care, termed handover, as a time of potential risk to patients.^8^,^9^,^10^,^11^ Thirty percent of residents on internal medicine call identified adverse situations which could have been predicted by, or benefited from better information in handover.^12^

The Royal College of Physicians and Surgeons of Canada (RCPSC) and the ACGME identify “communicator” as a core competency for training of residents including communication between physicians. In spite of this, most residents are not given formal education in handover skills. In a study in Northern Ireland it was found that only 13% of residents receive any explicit training in handovers and in the United States surveys have demonstrated that between 10% and 40% of programs provide formal training in handover.^7^,^8^,^9^ Reviews detailing mnemonics or standardized protocols for the transfer of patient care have been published previously.^15^,^16^,^17^ However, the literature is limited in the application and assessment of the effectiveness of these protocols. In addition, despite the important role handover may have in patient safety, there is no consensus on the educational methods to successfully teach it.

The objective of the current study was to systematically review the literature regarding education models available to teach handovers skills to healthcare professionals.

## Methods

### Criteria for considering studies for this review

#### Types of studies

Intervention studies, survey questionnaires, qualitative studies, descriptive studies and educational interviews.

#### Types of participants

The target population consisted of adult healthcare professionals, including physicians, nurses, residents, medical students and paramedic personnel. All healthcare professionals involved in the care of patients and in the transfer of patient information (handover) to a fellow professional were eligible for inclusion.

#### Types of interventions

The interventions of interest were education tools and resources available to teach handover to residents.

#### Types of outcome measures

##### 1) Primary outcomes

The primary outcome was a change in handover skill or efficiency, based on personal perception or external evaluation.

##### 2) Secondary outcomes

- Identification of strengths and weaknesses of current handover skills
- Healthcare personal satisfaction – Personal satisfaction with healthcare work.

### Search methods for identification of studies

#### Electronic searches

A systematic review was conducted of the English-language literature to identify publications of educational resources on patient handover skills. Medline, Embase, Health and Psychosocial Instruments (HaPI), Cumulative Index to Nursing and Allied Health Literature (CINAHL), science/social science citation index and PsychINFO databases were searched for English-language publications from January 1990 to December 2009. The search terms used were training tool, teaching module or combinations and variations and the MeSH terms “Professional Competence”, “Education” and “Teaching Methods and shift change, sign over, sign-out, handoff, handover or transfer” and the MeSH heading “Shift Reports”. References listed in papers included for data extraction (below) were reviewed to identify resources which may have been missed. The Medline search string is included as Appendix A.

#### Study Selection

Two investigators (M.M. and R.G.) independently reviewed the identified publications for inclusion or exclusion. An independent third reviewer (M.G.) resolved any disagreement. Papers were included if they contained an educational resource for teaching patient handover from any health professional group in any type of patient population. Exclusion criteria included handover in non-healthcare settings.

#### Data Extraction and Evaluation of Quality

Papers were then reviewed by one of two authors (M.M. or R.G.). The following information was extracted: profession or specialty targeted, country, educational model(s) presented, any outcome data, which may have been presented.

In addition to the classical 4-point rating scale for the levels of evidence in quantitative research^33^, we used a rating scale described by Cote and Turgeon to assess the level of evidence in the included qualitative studies^34^. This scale allows studies to be scored out of 12. In order to match the 4-point rating scale used for quantitative studies^33^, we reported scores of 1–2 as level 5, 3–4 as level 4, 5–7 as level 3, 8–10 as level 2 and 11–12 as level 1. Therefore, the lower the score out of 5, the greater the level of evidence and methodological quality. Further, where studies included an evaluation of an educational program they were assessed using the Kirkpatrick levels^35^, which describes the depth to which a program is evaluated.

## Results

### Results of the search

**Figure 1:**
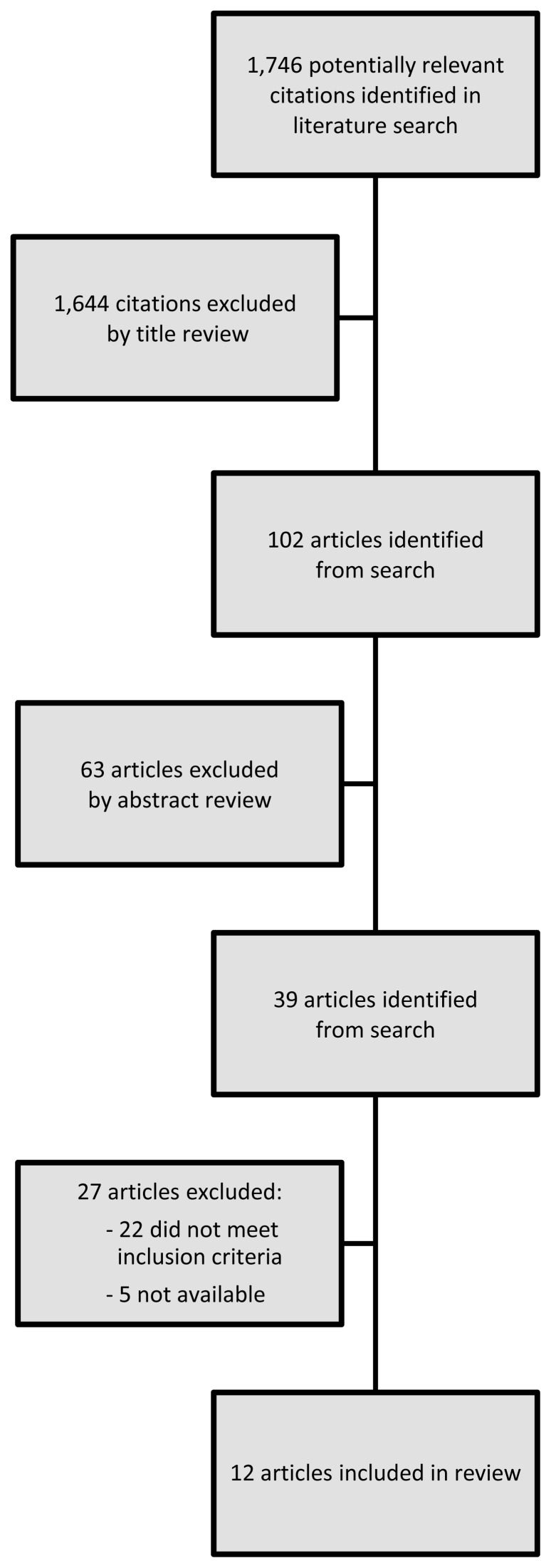
Diagram demonstrating article selection method

1746 studies were identified following our electronic search, of which 1644 were excluded following title review. The remaining 102 studies underwent abstract review and a further 63 papers were excluded. Of the remaining 39 studies, five could not be obtained and an additional 22 were excluded upon manuscript review (Figure 1): 5 were opinion pieces or editorials, 14 did not present an education model, and 3 did not provide details. Hand searching of references of the 12 publications yielded no additional studies that had not previously been investigated.

The 12 included studies are summarized in Table 1. Five publications were from the United States, five were from the United Kingdom, one was from Israel and one was from Australia. There were no articles identified from Canada. Five educational resources were directed toward residents alone, a further three towards a diverse group of professionals including physicians and physician trainees, and four were toward allied healthcare professionals.

Eight of the twelve included studies were evaluated using Kirkpatrick levels, as shown in Table 1. Seven of the nine studies were level 2 or 3 and one at level 1. There were no studies that investigated the effect on patient outcomes, which would be level 4. The overall quality of the included studies was relatively low, rated at 4 or 5, excluding the study by Cleland et al.,^30^ which was given a quality rating of 1.

Seven studies utilized simulation skills to teach handover.^21^,^22^,^23^,^30^,^32^ Simulation included a wide variety of methods ranging from role-playing to a re-created step-down unit with a simulated patient.^23^ Seven studies utilized observed handover, either in person or with video-tape review.^18^,^19^,^20^,^21^,^22^,^26^,^30^,^32^ Commonly this was done in conjunction with a simulation-training model. For example, Berkenstadt et al. used a simulation based communication workshop in a critical care setting to assess handover among nurses.^23^ These authors reported significantly improved reporting of patient information and physiologic parameters following the intervention. In the study by Nestel et al., role-playing was noted to be challenging. Specifically, perioperative practitioners experienced some difficulties playing the role of the consultant or senior physician. However, this experience improved the participating physicians’ understanding of what information was required for handover to be completed effectively.^18^

Eight studies used or suggested formal didactic sessions to teach handover skills.^15^,^18^,^19^,^21^,^22^,^24^,^25^,^31^ Chu et al. implemented a structured handover process with an initial one-hour didactic teaching session for internal medicine interns.^19^ This study included both observed interviews by experienced preceptors with feedback and a formal lecture. They reported that 85% of interns appreciated the supervised sessions, while only 18% appreciated the didactic sessions.^19^

Handover has been identified as a time for teaching professionalism.^15^,^20^ Two studies asserted that professionalism was an integral part of handover; they note that this is a time when “ownership” of a patient can be encouraged.^15^,^21^ Specifically, Arora et al. suggest that professionalism can be redefined with a focus on “shared responsibility”. This concept suggests that high standards of professionalism be maintained even if a long-standing relationship with the patient does not exist, such as during handover of the patient to a new physician.^15^ Cosgrove et al. suggest that a combination of lectures, simulation, case-based discussion and a move toward competency-based training may be needed.^21^

At least one study demonstrated handover skills being taught as part of a larger curriculum about patients.^22^ In study by Horwitz et al., a new curriculum using the “SIGN OUT” mnemonic and a combination of lectures and role-play was implemented.^22^ These authors reported that residents had greater confidence and comfort with handover after the curriculum. Overall the perceived usefulness of the structured oral handover format was rated 4.46 out of a possible 5.

In the study by Chu et al., the need to develop faculty expertise was identified.^19^ They utilized a combination of literature review and practice sessions with attending physician peer feedback to develop teaching capacity within the department.

Of the included studies four describe educational models without investigating effects on behavior or outcomes.^15^,^20^,^21^,^30^ Specifically Arora et al. provided a theoretical construct focusing on developing specific competencies, including effective communication, professionalism and handover education and evaluation.^15^ Klaber et al. highlighted key elements of handover, and the need to model them in a clinical setting.^20^ They also suggested the use of peer or video review of handover with reflection.

Two studies used third-party observation to describe behavioral changes.^23^, ^26^ Catchpole et al. implemented a handover protocol adapted from Formula 1 racing, and compared pre and post intervention handover in an intensive care (ICU) setting.^24^ In this study, a single observer was present for all handovers, and a reduction in technical errors and omitted information was reported. The remainder used questionnaires of student perception to evaluate either participant reaction (Kirkpatrick level 1) or impact (Kirkpatrick level 2) on learning. All studies that measured outcomes did so by questionnaire or observation after the intervention. No studies used randomization or control groups.

None of the studies have directly investigated the impact of handover education on patient mortality or morbidity, which would represent the highest level (4), of program evaluation in the Kirkpatrick model. All studies that investigated the transfer of information showed reduced errors of omission^23^,^24^,^25^,^26^ and one study showed improved rates of checks on critical machinery and medications with a decrease from 5.42 to 3.15 events per handover.^24^ The study by Catchpole et al. was the only study to investigate the length of handover and showed a non-significant decrease in the length of time for handover after a brief workshop and implementation of a handover protocol.^24^ The outcomes of the included studies are summarized in Table 2.

## Discussion

In spite of handovers being increasingly a part of a physician’s duty, there is limited research on educational models to teach handover skills to residents. We were able to identify only 12 articles, which dealt with the education of handover. Much of the literature on handover simply presents a mnemonic or checklist for handover without describing educational models.^27^,^28^,^29^

Although there is limited literature, the existing data indicate that there are methods of teaching handover that improve information transmission,^23^,^24^,^25^,^26^ healthcare provider comfort,^12^,^22^ and objective measures of errors.^24^ The majority of educational models demonstrating these benefits had defined goals for handover or a summary tool targeted to the work environment, used simulation under supervision of an experienced educator who provided direct feedback with the opportunity to practice learned skills. As well, direct supervision of handover by preceptors educated in handover models was identified as useful by resident physicians. Although there are no studies detailing improved patient safety directly, teaching handover practices improved resident confidence in their handover,^22^ and receiving residents are more comfortable with their duties at night when receiving adequate handover.^12^

In addition to patient safety, increased number of handovers and reduced physician work hours have led to concern over loss of professionalism or a sense of responsibility for and ownership of the patient’s care.^30^ This is identified as an agency problem in social science theory and handover is specifically identified as an opportunity to address this problem through fostering a culture of professionalism and responsibility.^15^,^20^

This study is limited to English language data, and by the paucity of existing literature. In order to expand the available literature we also included data from other professions, which may not be directly relevant to physicians. We were unable to identify Canadian literature on handover education. None of the studies investigated patient outcomes, and most of the studies that did investigate outcomes only measured provider perceptions of handover quality, which may not translate into benefits for patients. Furthermore, with high heterogeneity among the included studies, including both quantitative and qualitative methods, methodological assessment was limited. The quality of a systematic review is limited by the quality of the primary studies which were almost uniformly of low quality. However, the inclusiveness of this systematic review allows an overview of the available evidence, and highlights the need for further research in this field.

### Conclusion

In spite of a paucity of literature, most notably the absence of any literature which demonstrates changes in patient outcomes, the published literature demonstrates that there are models of education that can improve handover communication. This can improve inclusion of key elements in handover and make physicians more comfortable with handover. Further research on appropriate models of handover education is needed, both comparing different methods of education and evaluating the effect on patient safety. A cohort comparing observed interactions with simulation training on patient safety would provide valuable insight to direct future development of educational models.

## Figures and Tables

**Table 1:** Characteristics of included studies

Author, Year	Country	Target Audience	Type of Study	Educational Method	Educational Protocol	Kirkpatrick Level	Level of Evidence^33^,^34^
Arora, 2008	USA	Residents, IM	Descriptive	Theoretical Construct	Suggests 1) didactic teaching sessions on handover and 2) espousing a culture of professionalism during handover.	N/A	5
Chu, 2009	USA	Interns, Residents, OB, ER, IM	Survey	Didactic Teaching	One hour didactic session followed two handovers observed by preceptors with special training.	3	4
Cleland, 2009	UK	Residents, night nurse practitioners	Focus Groups	N/A	Suggest reflection on observed handovers and simulation with realistic situations, i.e. multiple patients and imperfect conditions.	N/A	1
Cosgrove, 2005	UK	Physicians, nurses, paramedics	Prospective Case series	Training course	2-day course in transfer of critically ill patients. Included lectures, small group sessions with simulation and case based discussion. Pre- and post tests for evaluation.	2	4
Horwitz, 2007	USA	Residents, IM	Survey	Training Session	Facilitated discussions to develop new curriculum using “SIGN OUT” mnemonic. 1h session including a demonstration, role-play with group feedback. Supported by a website, pocket card and page in interns manual.	N/A	5
Klaber, 2009	UK	Residents, Peds	Review, Opinion	Highlight Key Elements of Handover	Discusses need to model behavior in handover, provide leadership and direction, and value the handover and contribution of others. Suggest peer or video review and reflection.	N/A	5
Berkenstadt 2008	Israel	Nurses	Prospective Case series	Simulation based training	Analysis of critical event led to development of handover curriculum. Handover checklist and training in a high fidelity simulation centre with realistic situations, videotaping with review and debriefing.	3	4
Nestel, 2005	UK	Peri-operative specialists	Qualitative Survey	Teaching Session	2 hours session using discussion of theoretical materials, role-play with video taped review and written reflection. Built around objectives of: improvements in identification and application of presentation skills, and awareness of strengths and weaknesses among participants.	2	5
Catchpole, 2007	UK	Residents, AN	Prospective Interventional Study	Handover protocol	Brief workshop with a new structured handover protocol with defined roles and memory cards for key information. Debriefing sessions through case management rounds.	3	5
Clark, 2009	Australia	Nurses	Survey	Communication tools	Workshop with lecture and role-play on assertive communication and patient assessment with a handover prompt card and handover template using SBAR format. Utilized on unit “Champions” for monitoring and implementation.	1	5
Klamen, 2009	USA	Medical Students	Case series	Online Curriculum & Simulation based training	Online written and video curriculum with information on handovers, practice in tutorial groups and simulated handover on inpatient unit.	2	4
Iedema, 2009	USA	Physicians, Residents, Nurses	Interviews	Videotaped reflective learning	Video-reflexive learning and evolution of a handover approach. Handovers are taped and reviewed by staff and modifications incorporated into practice.	3	4

**Table 2:** Outcomes of included studies

Author, Year	Primary Outcome Measured	Current Weaknesses in Handover	Current Strengths in Handover	Healthcare Personal Satisfaction
Arora, 2008 (15)	N/A	Lack of standard instructional materials Lack of an assessment system		
Chu, 2009 (19)	85% of interns found supervised sessions useful, 18% appreciated the didactic session.	Need to improved accuracy of written handover Lack of digital program	Performing handover at same time and place on a daily basis Standardization of handover Presence of supervised handover during initial learning phase	84% of interns thought the overall program was useful. Overall high satisfaction among residents.
Cleland, 2009 (30)	N/A	Lack of structure to handover Lack of protected time for handover large number of patients to handover		Doctors and night nurse practitioners supported the concept of formal teaching of handover
Cosgrove, 2005 (21)	“Improvements” noted in handover and documentation.			
Horwitz, 2007 (22)	Residents reported greater confidence with sign out skills. Increased comfort with oral sign-out after training (3.94/5 vs. 3.27/5, p < 0.001).			
Klaber, 2009 (20)	N/A	Lack of formal teaching of handover Need clear objectives for handover		
Nestel, 2005 (18)	8/11 participants achieved all objectives. Survey indicated role-play challenging but rewarding.			Practitioners appreciated the different roles and perspectives during handover.
Berkenstadt 2008 (23)	Improved rates of handover of events during shift (from 88% to 100%), treatment goals (from 43% to 69%). Also improvements in basic information, checks on ventilator settings and medication.			
Catchpole, 2007 (24)	Technical errors (i.e. equipment not ready, alarms not on) decreased from 5.4 to 3.2 per handover and information omissions decreased from 2.1 to 1.1. Non-significant reduction in length of handover.			
Clark, 2009 (25)	Improvement from 32 to 68% receiving handover information needed, 68% noting improved handover after the intervention and 70–80% feeling more confident in communication skills.			72% of nurses agreed that they communicate more effectively following handover training
Klamen, 2009 (31)	Received positively by students (mean 4.2 out of 5), 38 of 69 made a medical error in the scenario.			Students generally satisfied with learning the process of handover.
Iedema, 2009 (32)	In interviews “all participants expressed satisfaction”. Some practitioners were noted to maintain reflexivity after the intervention.	Lack of standardized handover. Lack of handover at patients bedside		

## Appendix A Medline search string

**Ovid MEDLINE(R)** 1950 to January Week 4 2010

**Table:**

#	Searches	Results	Search Type
1	Patient Transfer/	4269	Advanced
2	(patient: adj2 transfer:).mp.	7928	Advanced
3	handover:.mp.	210	Advanced
4	hand over:.mp.	497	Advanced
5	handing over:.mp.	67	Advanced
6	(hand adj2 over:).mp.	1054	Advanced
7	(handing adj2 over:).mp.	72	Advanced
8	(transfer: adj2 care:).mp.	428	Advanced
9	sign-out:.mp.	119	Advanced
10	sign out:.mp.	119	Advanced
11	signing out:.mp.	16	Advanced
12	hand off:.mp.	111	Advanced
13	handoff:.mp.	153	Advanced
14	signout:.mp.	17	Advanced
15	signover:.mp.	0	Advanced
16	sign over:.mp.	27	Advanced
17	signing over:.mp.	3	Advanced
18	shift chang:.mp.	715	Advanced
19	or/1–18	10590	Advanced
20	curriculum/or competency-based education/or “mainstreaming (education)”/or problem-based learning/	51741	Advanced
21	exp Teaching Materials/	81097	Advanced
22	teaching/or computer user training/or models, educational/or patient simulation/or problem-based learning/or programmed instruction as topic/or computer-assisted instruction/or remedial teaching/	54437	Advanced
23	education, medical/or education, medical, continuing/or education, medical, graduate/or education, medical, undergraduate/or clinical clerkship/or “internship and residency”/or teaching rounds/	107423	Advanced
24	professional competence/or clinical competence/	62826	Advanced
25	19 and 20	33	Advanced
26	19 and 21	53	Advanced
27	19 and 22	40	Advanced
28	19 and 23	150	Advanced
29	19 and 24	121	Advanced
30	(teaching adj tool:).mp.	693	Advanced
31	19 and 30	0	Advanced
32	teaching module:.mp.	155	Advanced
33	19 and 32	0	Advanced
34	training tool:.mp.	287	Advanced
35	19 and 34	0	Advanced
36	training module:.mp.	250	Advanced
37	19 and 36	0	Advanced
38	ed.fs.	176375	Advanced
39	19 and 38	258	Advanced
40	workshop:.mp.	19045	Advanced
41	19 and 40	18	Advanced
42	25 or 26 or 27 or 28 or 29 or 39 or 41	484	Advanced
43	limit 42 to (english language and yr=“1990-Current”)	420	Advanced
